# *Staphylococcus aureus* iron-regulated surface determinant B (IsdB) protein interacts with von Willebrand factor and promotes adherence to endothelial cells

**DOI:** 10.1038/s41598-021-02065-w

**Published:** 2021-11-23

**Authors:** Mariangela J. Alfeo, Anna Pagotto, Giulia Barbieri, Timothy J. Foster, Karen Vanhoorelbeke, Vincenzo De Filippis, Pietro Speziale, Giampiero Pietrocola

**Affiliations:** 1 Department of Molecular Medicine, Biochemistry Unit, University of Pavia, Viale Taramelli 3/b, 27100 Pavia, Italy; 2 Department of Pharmaceutical and Pharmacological Sciences, University of Padua, Via Marzolo 5, 35131 Padua, Italy; 3 Department of Biology and Biotechnology “Lazzaro Spallanzani”, University of Pavia, Via Ferrata 9, 27100 Pavia, Italy; 4 Microbiology Department, Trinity College Dublin, Dublin, Ireland; 5 Laboratory for Thrombosis Research, KU Leuven Campus Kulak Kortrijk, Kortrijk, Belgium

**Keywords:** Proteins, Bacterial pathogenesis

## Abstract

*Staphylococcus aureus* is the cause of a spectrum of diseases in humans and animals. The molecular basis of this pathogenicity lies in the expression of a variety of virulence factors, including proteins that mediate adherence to the host plasma and extracellular matrix proteins. In this study, we discovered that the iron-regulated surface determinant B (IsdB) protein, besides being involved in iron transport and vitronectin binding, interacts with von Willebrand Factor (vWF). IsdB-expressing bacteria bound to both soluble and immobilized vWF. The binding of recombinant IsdB to vWF was blocked by heparin and reduced at high ionic strength. Furthermore, treatment with ristocetin, an allosteric agent that promotes the exposure of the A1 domain of vWF, potentiates the binding of IsdB to vWF. Both near-iron transporter motifs NEAT1 and NEAT2 of IsdB individually bound recombinant A1 domain with K_D_ values in the micromolar range. The binding of IsdB and adhesion of *S. aureus* expressing IsdB to monolayers of activated endothelial cells was significantly inhibited by a monoclonal antibody against the A1 domain and by IsdB reactive IgG from patients with staphylococcal endocarditis. This suggests the importance of IsdB in adherence of *S. aureus* to the endothelium colonization and as potential therapeutic target.

## Introduction

*Staphylococcus aureus* is a leading cause of serious diseases such as sepsis and infective endocarditis^1^. The endothelium is a major target of endovascular infection and *S. aureus* has developed several mechanisms to attach to both endothelial cells lining the heart and blood vessel wall and also to the exposed extracellular matrix when the endothelium is damaged. For this purpose, the bacterium expresses a repertoire of cell wall-anchored (CWA) surface proteins that mediate adhesion to the tissue structures^2–4^. The archetype of such interactions is the bacterial binding to endothelium under flow via von Willebrand factor (vWF)^5^, a large glycoprotein produced by activated endothelial cells and megakaryocytes, the precursor cells of platelets. The mature 2050 amino acid long monomer consists of four homologous units which are arranged in the following sequence: D1–D2–D′–D3–A1–A2–A3–D4–B1–B2–B3–C1–C2–CK. Each domain can specifically bind several ligands. The D′D3 domain interacts with factor VIII, the A1 domain binds to the platelet GPIb receptor, heparin, and collagen type IV and VI, the A2 domain contains a cryptic cleavage site for ADAM13 protease, the A3 domain binds fibril-forming collagens I and III, and C1 is the binding domain for integrin αIIbβ3 and α_v_β_3_ through an RGD motif. In the intracellular environment, vWF is organized in compact multimers stored in organelles called Weibel-Palade bodies.

vWF multimerization is initiated by the formation of disulfide bonds between C-terminal domains of two protomers leading to tail-to-tail homodimerization followed by disulfide linkage between N-terminal domains of adjacent dimers. Multimers secreted in the plasma may contain up to 40 subunits and can reach a length of around several micrometers. They adopt a globular conformation under normal blood flow conditions. Under high shear flow, multimers undergo a conformational change from a compact to a stretched configuration eventually leading to the exposure of cryptic binding sites for platelet recruitment and extracellular matrix proteins such as collagen^6^.

At least two staphylococcal factors have been shown to interact directly with vWF, the CWA protein A (SpA)^7^ and the secreted coagulase vWF-binding protein (vWbp)^8^. Recently, a fibrinogen-binding protein named Vhp, showing significant amino acid identity to vWbp, has been identified in *S. aureus*^9^, but it remains to be determined whether Vhp binds to vWF. SpA, expressed both in iron-rich medium and iron starvation conditions^10^, comprises a tandem array of five separately folded three-helical bundles, each of which can bind several ligands: the interface between helices 1 and 2 binds the A1 domain of vWF^11,12^. SpA also binds the D’-D3 domain of vWF^12^ with lower affinity. vWbp is a multi-domain protein that interacts with a variety of ligands including prothrombin^13^, fibrinogen^14^, fibronectin, and Factor XIII^15^. Importantly, this protein also binds the A1 domain of vWF via a 26 amino acid sequence located in the C-terminal region^8^. Under shear stress, the binary complex consisting of vWF and vWbp associates with the cell wall-anchored clumping factor A (ClfA). The resulting ternary complex is extremely stable, resisting forces in the 2nN range and mediates the anchoring of *S. aureus* to the blood vessel wall^16^.

Adhesion of *S. aureus* grown in rich medium containing iron to host cells has been extensively studied. Conversely, the *S. aureus* pathophysiology *in vivo*, where the bacterium has restricted access to iron (the amount of free iron found within the serum is negligible, as it is usually complexed to high a affinity iron-binding protein)^17^, remains to be investigated. The lack of available iron *in vivo* leads to the upregulation of several genes including those encoding iron regulated-surface determinant (Isd) proteins. An important role of Isd proteins is to capture heme from hemoglobin (Hb) and transport it into the bacterial cell^17^. The Isd system comprises four surface proteins (IsdABCH), a membrane ABC transporter (IsdEF), and two intracellular heme-degrading enzymes (IsdGI)^18^. The IsdABCH proteins contain up to three copies of a *NEA*r iron *T*ransporter (NEAT) motif: a single NEAT domain is present in IsdA and IsdC, whereas IsdB and IsdH contain two and three NEAT modules, respectively. Regarding IsdB, no structural data at atomic resolution are available, although the crystallographic structure of IsdB-hemoglobin (Hb) complex^19^ indicates that IsdB has a dumbbell-like shape, with the two NEAT domains laying almost orthogonal to each other and joined by a highly flexible triple helix linker region^19,20^. Each NEAT domain adopts the characteristic eight-strand immunoglobulin-like β-sandwich fold with a central 3_10_-helix that forms a hydrophobic cavity. Notably, the two NEAT domains share low sequence identity (about 12%), in line with their known different molecular recognition properties. In fact, NEAT1 preferentially binds Hb, while NEAT2 is involved in heme extraction from the chains of Hb^19,20^.

Besides acting as hemophores, IsdA, IsdB, and IsdH of *S. aureus* are known to have other biological activities. IsdA binds human proteins including fibrinogen and fibronectin^21^, and is involved in the evasion of the host innate defenses in the skin^22^. IsdH plays a role in *S. aureus* escaping phagocytosis through the inactivation of opsonin C3b^23^. IsdB binds to integrin α_v_β_3_ expressed on endothelial cells^24^ and to integrin GPIIb/IIIa on platelets, and promotes staphylococcal adherence and internalization by non-phagocytic human cells^25^. Furthermore, IsdB acts as a receptor for the host protein vitronectin (Vn) and Vn binding mediates adherence to and invasion of HeLa and HUVEC monolayers^26^. In this study, we further investigated the binding of *S. aureus* cells to host proteins and discovered that IsdB interacts with vWF and its expression amplifies the vWF-dependent adhesion of *S. aureus* to endothelial cells. We also found that bacterial adhesion is blocked by anti-IsdB IgG isolated from patients with staphylococcal endocarditis, suggesting the possible use of immunological therapies to combat the *S. aureus* colonization/infection of the vascular system.

## Results

### Identification of a novel *S. aureus* vWF-binding protein expressed in iron-limited conditions

**Figure 1:**
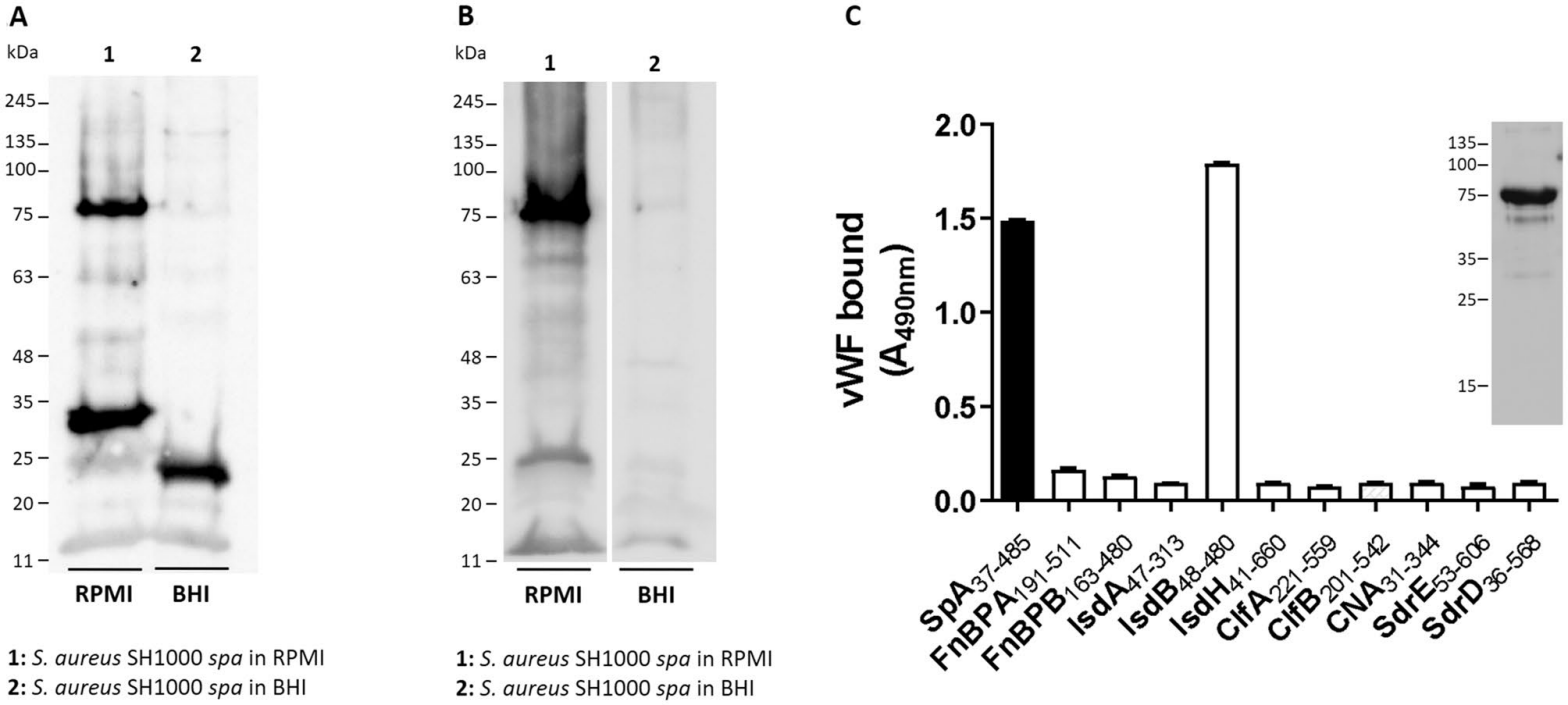
Identification of a vWF receptor in *S. aureus* SH1000 *spa*. **(A)** Lysostaphin-released material from *S. aureus* SH1000 *spa* grown in RPMI (lane 1) and BHI (lane 2) was subjected to Far Western Blotting. The nitrocellulose membrane was probed with human vWF followed by polyclonal rabbit vWF antibody and HRP-conjugated goat anti-rabbit IgG. **(B)** Lysostaphin-released material from *S. aureus* SH1000 *spa* grown in RPMI and BHI was subjected to Western Immunoblotting. The nitrocellulose membrane was probed with a rabbit anti-IsdB IgG and HRP-conjugated goat anti-rabbit IgG. Molecular mass standards are indicated on the left of the panels. **(C)** Binding of vWF to surface proteins from *S. aureus*. Microtiter wells were coated with purified recombinant A regions of the indicated CWA proteins of *S. aureus* and incubated with human vWF. Ligand bound to the wells was detected with a polyclonal rabbit vWF antibody followed by HRP-conjugated goat anti-rabbit IgG. The data points are the means ± SD of two independent experiments. In the inset, binding of the vWF to purified recombinant IsdB NEAT1-NEAT2 protein in a Western blotting assay is shown. The full-length blots are presented in Supplementary Fig. S3.

A property of *S. aureus*, shared with other Gram-positive bacteria, is the ability to use several different CWA proteins to interact with a specific host component. As an example of this redundancy, *S. aureus* can adhere to fibrinogen via clumping factors, ClfA^27^ and ClfB^28^, and fibronectin-binding proteins, FnBPA^29^ and FnBPB^30^. To date, the only CWA protein known to interact with vWF is SpA^7^. In the search for new *S. aureus* surface component(s) potentially involved in vWF binding, SH1000 *spa* deletion mutant cells were grown either in iron-rich (BHI) or iron-poor (RPMI) medium. They were digested with lysostaphin, and the released material was subjected to SDS-PAGE under reducing conditions and analyzed for vWF-binding by far Western blotting. A 75 kDa vWF-binding protein was detected in material released from cells grown in RPMI (Fig. 1A, lane 1), whereas no significant signal was detected in proteins originating from cells grown in BHI (Fig. 1A, lane 2) suggesting that binding depends on a protein that is induced by iron starvation. No detection of 75 kDa protein was observed with the anti-vWF IgG alone (data not shown). Additional low molecular weight molecules (25-30 kDa) were observed in the material released from bacteria grown in both media.

In iron starvation conditions, *S. aureus* expresses several iron-regulated surface proteins. Among these, IsdB with a molecular weight of 75 kDa is a potential candidate receptor for vWF. To validate this hypothesis, lysostaphin-solubilized cell-wall fractions of *S. aureus* SH1000 *spa* cells were subjected to SDS-PAGE and immunoblotting and probed with an IsdB antibody (Fig. 1B). A major signal of 75 kDa molecule was detected in the proteins released from cells grown in RPMI, indicating that IsdB is the possible vWF-binding protein (Fig. 1B, lane 1). No signal was observed in the material from cells grown in BHI (Fig. 1B, lane 2).

To confirm this, purified recombinant ligand-binding domains of several *S. aureus* CWA proteins were screened for the ability to bind vWF by an ELISA-type assay. In particular, vWF showed a binding activity for IsdB NEAT1-NEAT2 comparable to that already reported for SpA, whereas no binding to the N-terminal domains of the other proteins tested was detectable (Fig. 1C). The specificity of IsdB binding to vWF was confirmed by the finding that IsdA and IsdH did not interact with vWF.

The binding of vWF to recombinant IsdB was also observed by far Western ligand blotting (Fig. 1C, inset). Altogether, these data provide evidence that IsdB is a novel surface receptor of vWF.

### Capture of vWF by *S. aureus*

**Figure 2:**
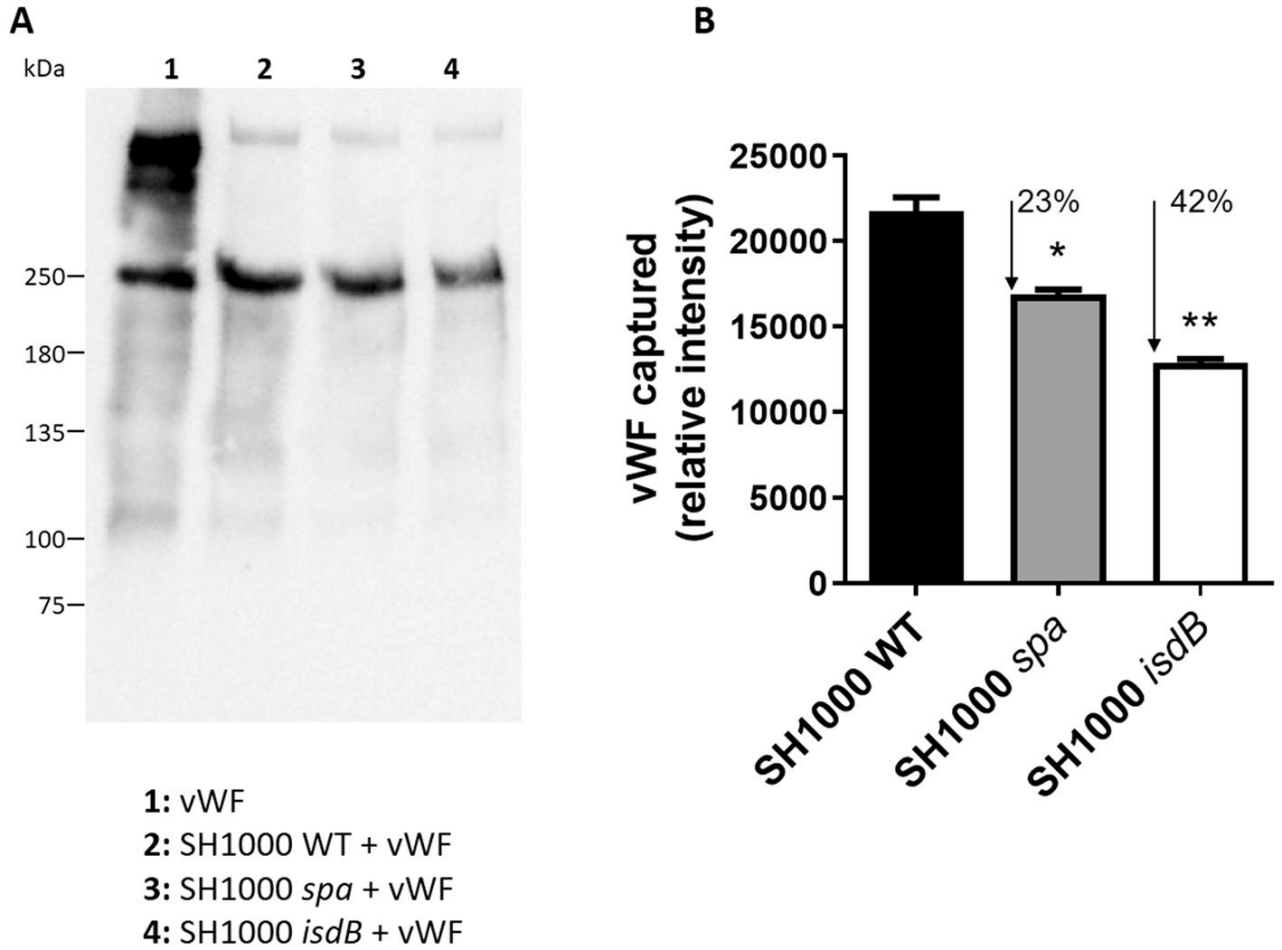
Capture of vWF *by S. aureus* cells. **(A)** *S. aureus* SH1000 WT and its *spa* or *isdB* mutant were grown in RPMI to stationary phase. The staphylococcal cells were incubated with purified human vWF (5 µg/ml) and bacteria-bound protein was released by extraction buffer and subjected to SDS-PAGE and Western Immunoblotting. 2 µg of purified vWF was loaded as control. The membrane was probed with a polyclonal rabbit vWF antibody followed by HRP-conjugated goat anti-rabbit IgG. Molecular masses of standard proteins are indicated on the left. The full-length blot is presented in Supplementary Fig. S3. **(B)** Densitometric analysis of vWF bound *to S. aureus* WT and its isogenic *spa* or *isdB* mutant. Statistically significant differences are indicated (*, *P* < 0.05; **, *P* < 0.01). The reported data are the mean values ± SD from three independent experiments.

To investigate the role of IsdB in promoting the recruitment of soluble vWF, *S. aureus* SH1000 WT and its isogenic *spa* or *isdB* mutants were grown in iron-deficient conditions, incubated with vWF, and the amount of ligand captured was quantified by Western blotting and densitometry. Isogenic *spa* or *isdB* mutants captured 23% and 42% less vWF than the wild type strain, respectively, suggesting that under static conditions IsdB and SpA together contribute about two-thirds to the overall vWF capture by *S. aureus* SH1000 and that other unidentified receptors could be involved in vWF-binding (Fig. 2A,B). The mutual involvement of each receptor in vWF capture would likely depend on the copy number of the protein expressed by a particular strain, the stoichiometry of the interaction as well as the environmental conditions (e.g., static versus dynamic conditions). Thus, the reduced contribution to the capture of vWF by the SH1000 WT could be related to its low expression of SpA.

When human plasma was used as a source of vWF no capture of vWF was observed (data not shown). This contrasts with our ability to detect IsdB-promoted capture of vitronectin from plasma^26^. This difference is likely to be due to the different plasma concentrations of vWF and Vn (10 μg versus 300 μg/ml).

### IsdB-mediated adhesion of bacteria to immobilized vWF

**Figure 3:**
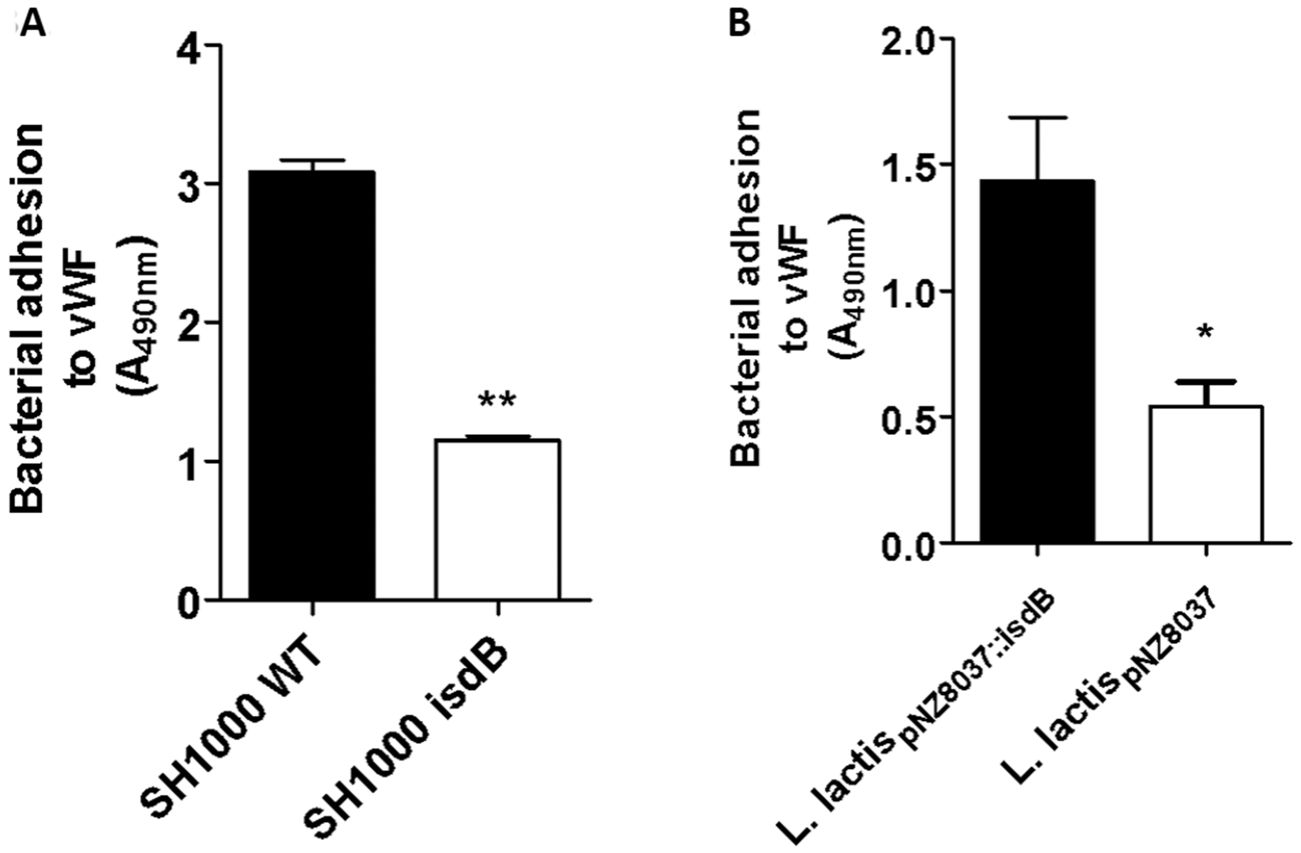
Adhesion of *S. aureus* and *L. lactis* ectopically expressing IsdB to immobilized vWF. (**A**) Adhesion of *S. aureus* SH1000 WT and its isogenic *isdB* mutant to immobilized vWF. Microtiter wells coated with vWF were incubated with cells of *S. aureus* SH1000 WT and its *isdB* mutant obtained from cultures grown in RPMI. Bacteria bound to vWF were detected by the addition of HRP-conjugated rabbit anti-mouse IgG to the wells. Statistically significant difference is indicated (**, P < 0.01). (**B**) Adhesion of *L. lactis* expressing IsdB to immobilized vWF. Microtiter wells coated with vWF were incubated with *L. lactis* ectopically expressing IsdB (*L. lactis *_pNZ8037::isdB_) or *L. lactis* carrying the empty vector (*L. lactis*_pNZ8037_). Bacteria bound to vWF were detected incubating the wells with a polyclonal rabbit anti-*L. lactis* IgG and HRP-conjugated goat anti-rabbit IgG. Statistically significant difference is indicated (*, P < 0.05). The data points are the means ± SD from three independent experiments, each performed in triplicate.

To evaluate the role of IsdB in bacterial adhesion to vWF, we compared the ability of SH1000 WT and its *isdB* mutant to bind immobilized vWF in an ELISA-type assay. A significantly higher attachment of the WT strain compared to the *isdB* mutant was observed, indicating the important role of IsdB in bacterial adhesion to vWF (Fig. 3A). The ability of IsdB to promote adhesion to vWF was also examined using the surrogate host strain *L. lactis* expressing IsdB from a gene cloned into the plasmid vector pNZ8037 (*L. lactis*_pNZ8037::*isdB*_). When lactococci were tested for adhesion to surface-coated vWF, significantly higher adhesion of *L. lactis*_pNZ8037::*isdB*_ was observed compared to that of *L. lactis* harboring the empty vector (Fig. 3B).

### Interference of vWF binding ligands on the interaction of IsdB with vWF

**Figure 4:**
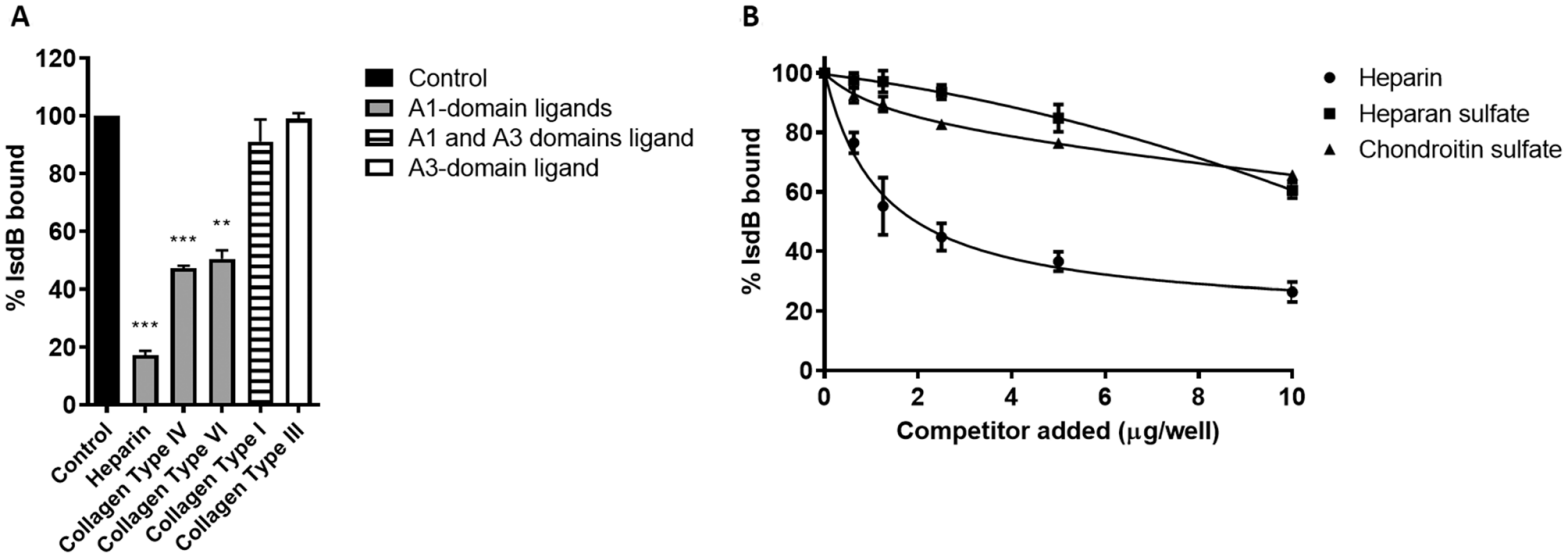
Interference of vWF ligands with the VWF/IsdB interaction. **(A)** the effect of vWF ligands on the binding of IsdB NEAT1–NEAT2 to immobilized vWF. Microtiter wells coated with vWF were incubated with IsdB NEAT1–NEAT2 in the presence of the indicated potential competitors. IsdB NEAT1-NEAT2 binding to the wells was detected by the addition of a polyclonal rabbit anti-IsdB IgG followed by HRP-conjugated goat anti-rabbit IgG. Binding data are expressed as a percentage of the control, i.e. incubation performed in the absence of any potential IsdB competitor. Statistically significant differences are indicated (**, P < 0.01, ***, P < 0.001). **(B)** The effect of polyanionic compounds on the binding of IsdB NEAT1–NEAT2 to immobilized vWF. Microtiter wells coated with vWF were incubated with IsdB NEAT1–NEAT2 in the presence of increasing concentrations of heparin, heparan sulfate, and chondroitin sulfate. Binding was detected as reported in **(A)**. The data points are the means ± SD from three independent experiments, each performed in triplicate.

A competitive inhibition assay was developed to investigate the effect of heparin and other specific vWF-ligands (collagen type I, type III, type IV, and type VI)^31,32^ on the binding of IsdB to vWF. Heparin and collagen type IV and VI, each of which interacts with the A1 domain, reduced IsdB binding to vWF by 80% and 50%, respectively. In contrast, collagen type I, which binds to the A3 domain, showed a marginal inhibitory effect (Fig. 4A). We also compared the dose-dependent inhibition of IsdB binding to vWF by the glycosaminoglycans, including heparin, heparan sulfate, and chondroitin sulfate. Unlike heparan sulfate and chondroitin sulfate, heparin inhibited the IsdB-vWF interaction up to 80% at the highest concentration (Fig. 4B). A further clue to the involvement of the A1 domain in IsdB binding came from the treatment of vWF with ristocetin, an allosteric effector that promotes elongation/stretching and exposure of cryptic regions including the A1 domain^33^. Ristocetin induced a threefold higher reactivity of immobilized vWF with the anti-A1 domain mAb 6D1^34^ as compared to the untreated protein in an ELISA assay (Fig. S1A). The binding of ristocetin-treated vWF to immobilized IsdB was increased by nearly 40% compared to the binding of untreated vWF (Fig. S1B). Together these results indicate that IsdB binds the A1 domain of vWF.

### Localization of binding sites in IsdB and vWF and affinity studies

**Figure 5:**
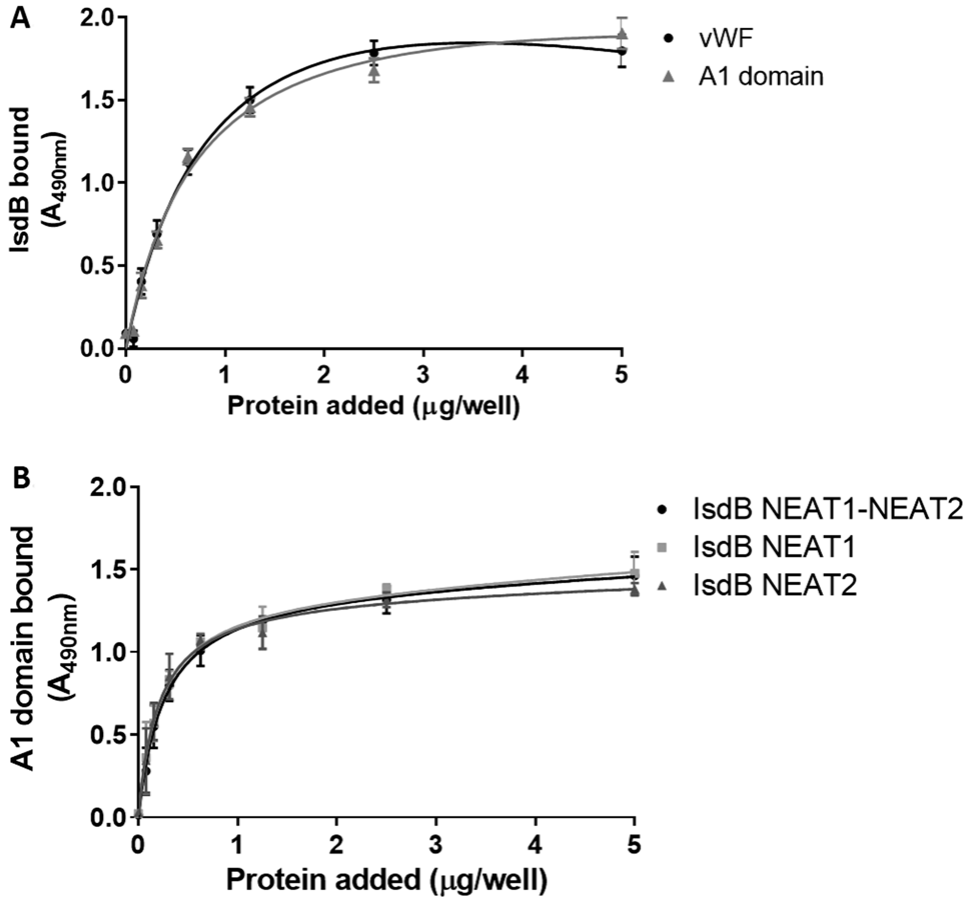
Localization of the binding sites involved in the interaction of IsdB with vWF. **(A)** Concentration dependent-binding of IsdB NEAT1-NEAT2 to immobilized vWF or the vWF A1 domain. vWF and the recombinant A1 domain were immobilized onto microtiter wells and incubated with increasing concentrations of IsdB NEAT1-NEAT2. Complex formation was detected by addition to the wells of a polyclonal rabbit anti-IsdB IgG followed by HRP-conjugated goat anti-rabbit IgG. **(B)** Binding of the A1 domain to IsdB proteins. IsdB NEAT1–NEAT2 protein and its NEAT1 and NEAT2 subregions were immobilized onto microtiter wells and incubated with increasing concentrations of the A1 domain. Ligand binding was detected as reported in Fig. 1C. The data points are the means ± SD from three independent experiments, each performed in duplicate.

To directly show that the A1 domain of vWF carries the IsdB-binding region, the isolated A1 domain of vWF was expressed in *E. coli* and tested by ELISA-type binding assays (Fig. 5). First, the interaction between immobilized vWF or the A1 domain and IsdB NEAT1-NEAT2 was examined. IsdB bound to the A1 domain in a saturable and dose-dependent manner and the binding profile appeared to be very similar to that obtained for the binding of IsdB to the full-length vWF (Fig. 5A). This implies that there is a single binding site for IsdB in vWF. To map the vWF-binding region(s) within the IsdB protein, the binding of the A1 domain to immobilized IsdB NEAT1-NEAT2 and the IsdB NEAT1 or IsdB NEAT2 modules was assessed. Our data indicate that the A1 domain bound dose-dependently and saturably to each recombinant module and with a binding pattern resembling that exhibited by IsdB NEAT1-NEAT2 (Fig. 5B).

**Figure 6:**
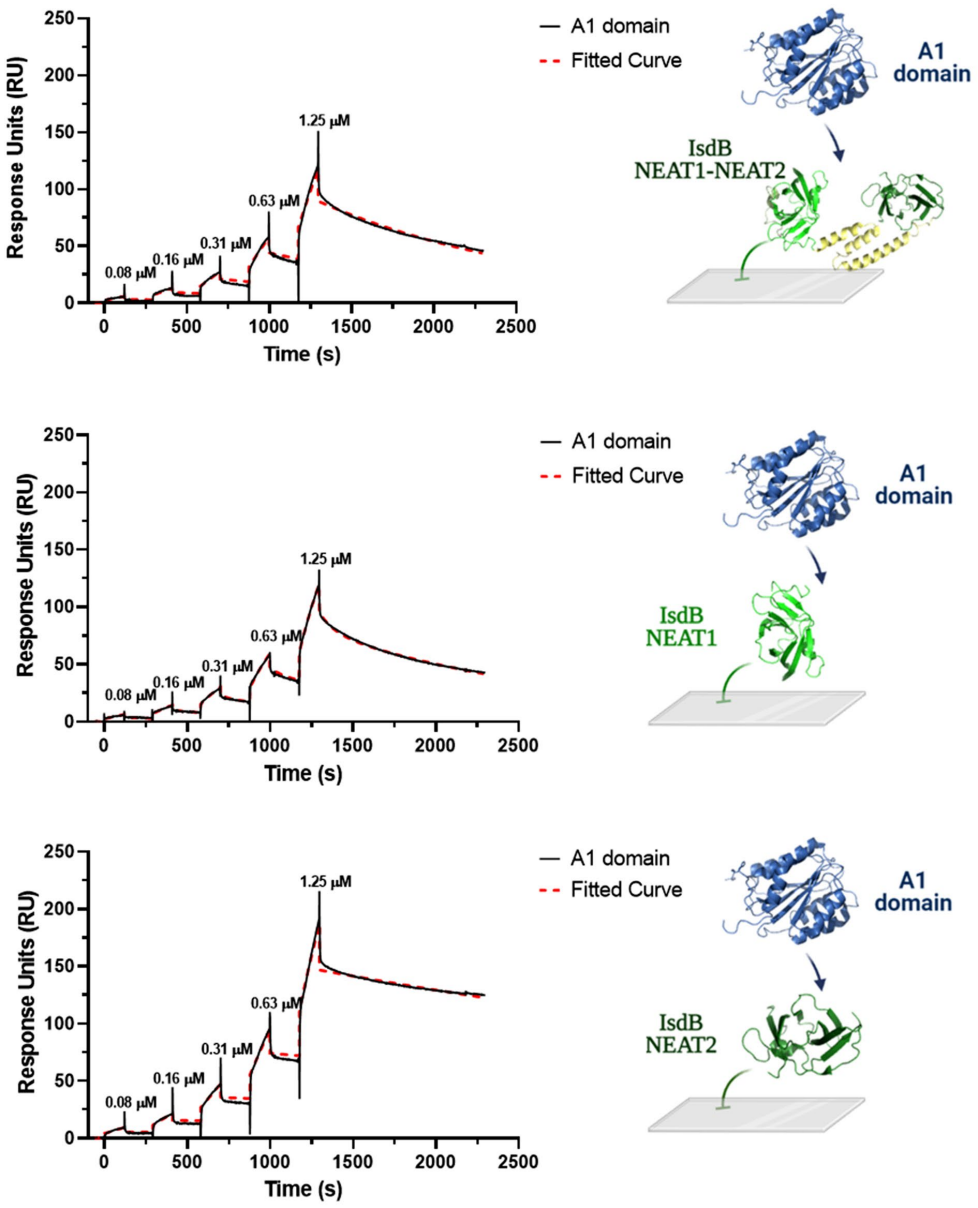
BIAcore analysis of A1 binding to IsdB proteins. 6xHis-tagged IsdB NEAT1-NEAT2 or isolated NEAT1 and NEAT2 were immobilized on a NTA-Ni^2+^ sensor chip, while increasing concentrations of recombinant A1 (0–1.25 μM), lacking the 6xHis tag, were loaded in the mobile phase, following the single-cycle operation mode. Sensorgrams were subtracted from reference flow cell data and then fitted with the equation describing the heterogeneous ligand binding model to obtain the values of K_D_ and RUmax for the two ligand populations considered (L1 and L2) along with their relative abundance. IsdB NEAT1–NEAT2: K_D1_ = 1.7 ± 0.3 μM; K_D2_ = 0.9 ± 0.2 μM; RU_max1_ = 354 ± 21; RU_max2_ = 355 ± 23; L1 and L2 = 50%. NEAT1: K_D1_ = 53 ± 2 μM; K_D2_ = 5.3 ± 0.3 μM; RU_max1_ = 948 ± 40; RU_max2_ = 2705 ± 125; L1 = 26%, L2 = 74%. NEAT2: K_D1_ = 0.3 ± 0.1 μM; K_D2_ = 0.4 ± 0.1 μM; RU_max1_ = 529 ± 51; RU_max2_ = 527 ± 39; L1 and L2 = 50%. All measurements were carried out at 25 °C, in PBS containing 0.005% v/v Tween 20.

To determine the affinity of the A1 domain for IsdB proteins by surface plasmon resonance (SPR), IsdB NEAT1-NEAT2 and the single NEAT domains were immobilized on an NTA-Ni^+2^ sensor chip and incubated with increasing amounts of A1 domains (from 0.08 to 1.25 μM) in a single-cycle operation mode. The sensorgrams shown in Fig. 6 revealed a complex binding system and were best fitted within the framework of a 1:1 stoichiometric heterogeneous ligand binding model^35,36^, whereby a single molecule of the A1 domain binds to a single site on IsdB proteins which, after noncovalent capture on the NTA-Ni^+2^ sensor chip, can assume different orientations on the chip surface. From this analysis, dissociation constant (K_D_) values in the low micromolar range for the A1/IsdB protein complexes were obtained (see the legend to Fig. 6). However, the A1 domain showed a higher affinity for the NEAT2 domain than that observed for the IsdB NEAT1-NEAT2 or NEAT1 domain. Comparative analysis of the affinities of IsdB NEAT1–NEAT2 and NEAT1 and NEAT2 revealed a non-additive behavior of the isolated domains in binding to the A1 domain. More specifically, the affinity of IsdB NEAT1-NEAT2 for A1 is much lower than the sum of the affinities of isolated NEAT1 and NEAT2. This finding is suggestive of a different binding mechanism that the two NEAT domains likely exploit for interacting with A1 in the isolated form or when they are embedded in the single-chain IsdB NEAT1-NEAT2 protein.

### Electrostatic properties of IsdB and A1 domain

To determine whether ionic forces play a role in the interaction of IsdB with vWF, the effect of increasing NaCl concentrations on IsdB binding was assessed. The addition of salt significantly reduced the binding of IsdB to immobilized vWF. At 0.5 M NaCl IsdB binding was reduced by > 70% (Fig. S2A). These results provide indirect evidence that charge-charge interactions play a role in IsdB-vWF complex formation.

Electrostatic potential calculations (Supplementary Fig. S2B, panels a—d), obtained from the crystallographic structure of IsdB deduced from the IsdB/Hb complex^19^, indicate that the linker region is highly electropositive whereas the NEAT domains display an asymmetric distribution of charges. This is more pronounced in NEAT2, where a predominantly negative face could be identified. On the other hand, NEAT1 is mainly electropositive, with some interspersed negative spots encompassing the β-strand 153–156 and α-helix 163–169.

The A1 domain has a cuboid shape, with a central hydrophobic parallel, eight-stranded parallel β-sheet flanked by three α-helices on each side of the β-sheet^37^. Electrostatic potential calculations carried out on the A1 domain (Supplementary Fig. S2B, panels e—h) identified two distinct faces of opposite charges, i.e. a strongly electropositive face covering helices α4 and α5 and an electronegative face encompassing helices α1 and α7. Thus, the highly charged nature of both IsdB and A1 suggests that ionic forces may play an important role in macromolecular complex formation. The steric and electrostatic complementarity of the IsdB and A1 structures (Supplementary Fig. S2B) suggest that the highly electropositive face of the globular A1 domain can preferentially couple with the electronegative surfaces in the concave dumbbell structure of IsdB NEAT1-NEAT2, as observed in the X-ray structure of the IsdB-hemoglobin complex^19^.

### IsdB binding to vWF mediates adhesion of *S. aureus* to endothelial cells

**Figure 7:**
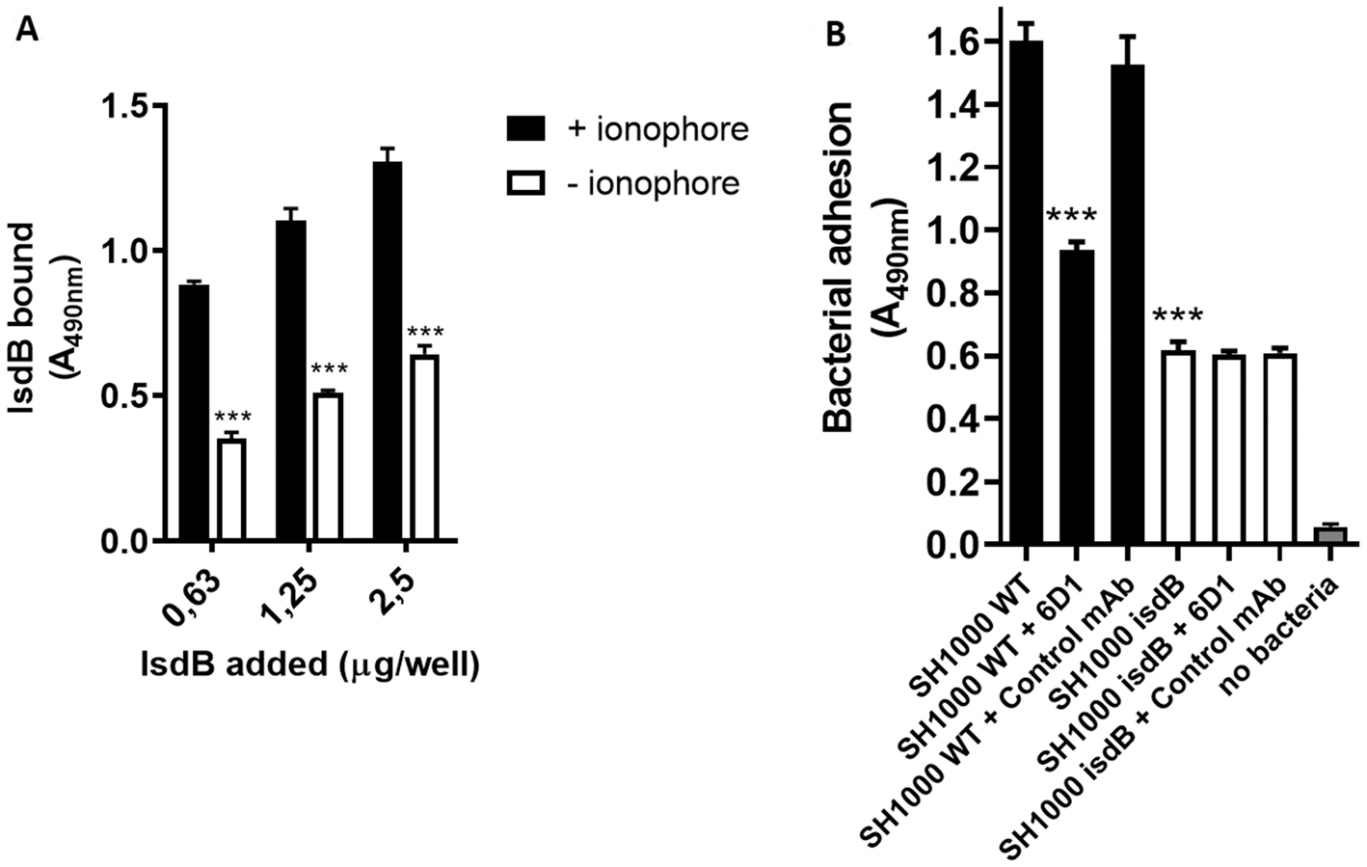
IsdB-mediated adhesion to HUVEC cell monolayers. **(A)** Confluent umbilical vein endothelial cell (HUVEC) monolayers were incubated with the calcium ionophore A23187 and fixed with paraformaldehyde. The monolayers were incubated with the indicated amounts of IsdB NEAT1-NEAT2 and binding of the ligand to the cells was detected by addition to the wells of a rabbit polyclonal IsdB antibody followed by an HRP-conjugated goat anti-rabbit IgG. The binding of IsdB NEAT1-NEAT2 to the ionophore-untreated cells is also reported. Statistically significant difference is indicated (***, P < 0.001). **(B)** Endothelial cell monolayers treated as reported in A were incubated with *S. aureus* SH1000 WT and isdB mutant bacteria. Adherence to the monolayers was detected by the addition of HRP-conjugated rabbit anti-mouse IgG to the wells. The effect of antibodies on the adhesion of bacteria to endothelial cells was tested by incubating *S. aureus* cells with monolayers in the presence of an anti-A1 6D1 antibody or an isotype control mAb. Binding of HRP-conjugated rabbit anti-mouse IgG to monolayers in the absence of bacteria is also reported. Statistically significant difference is indicated (***, P < 0.001). Error bars show S.D. of the means from three independent determinations, each performed in triplicate.

Endothelial cells (ECs) and megakaryocytes are the only cells that synthesize vWF. Thus, we asked whether *S. aureus* expressing IsdB can adhere to vWF associated with the ECM of ECs. Increasing amounts of IsdB NEAT1–NEAT2 were incubated with monolayers of human umbilical vein endothelial cells (HUVEC) pre-treated with the calcium ionophore A23187, a compound that induces the fusion of Weibel-Palade bodies with the plasma membrane and secretion of vWF. IsdB bound dose-dependently to activated cells and significantly more than to untreated cells (Fig. 7A). The mechanism of the IsdB binding to resting endothelial cells is unknown.

The adhesion of the SH1000 WT and its isogenic *isdB* mutant to activated HUVEC cells was explored using a bacterial detection method based on the recognition of SpA by a HRP-conjugated rabbit IgG. The WT strain showed a level of adhesion to the monolayers nearly three times higher than the *isdB* mutant (Fig. 7B), indicating the active involvement of IsdB in the process. To exclude the possibility that the different levels of adhesion by WT and its *isdB* mutant to monolayers could be due to the differential expression of IgG-binding proteins, the SH1000 strain and the its isogenic *isdB* mutant were immobilized onto microtiter wells and then probed with HRP-labelled rabbit IgG. As reported in Fig. 3SA, both bacteria captured similar amounts of antibody, suggesting that they express the same level of protein A and/or other IgG-binding proteins. To rule out a potential interference in the assay due to a presumptive IgG-binding activity of IsdB, we also demonstrated that IsdB does not bind the HRP-conjugated IgG or the anti-*L. lactis* antibody (Fig. 3SB).

To evaluate the role of the A1 domain of vWF in promoting adherence of *S. aureus* to HUVEC cells via IsdB, a bacterial adhesion assay was performed in the presence of mAb 6D1. The mAb 6D1, but not an isotype control mAb against ClfA, inhibited binding of IsdB to the A1 domain by 55% at a concentration of 250 ng/well (Fig. 4S), and significantly decreased the adhesion of the WT strain to activated HUVEC cells (Fig. 7B). Conversely, no effect by the mAb 6D1 on adhesion of SH1000 *isdB* mutant to the monolayers was observed.

### Effect of IgG from patients with infective endocarditis on adhesion of *S. aureus* to HUVEC cells

**Figure 8:**
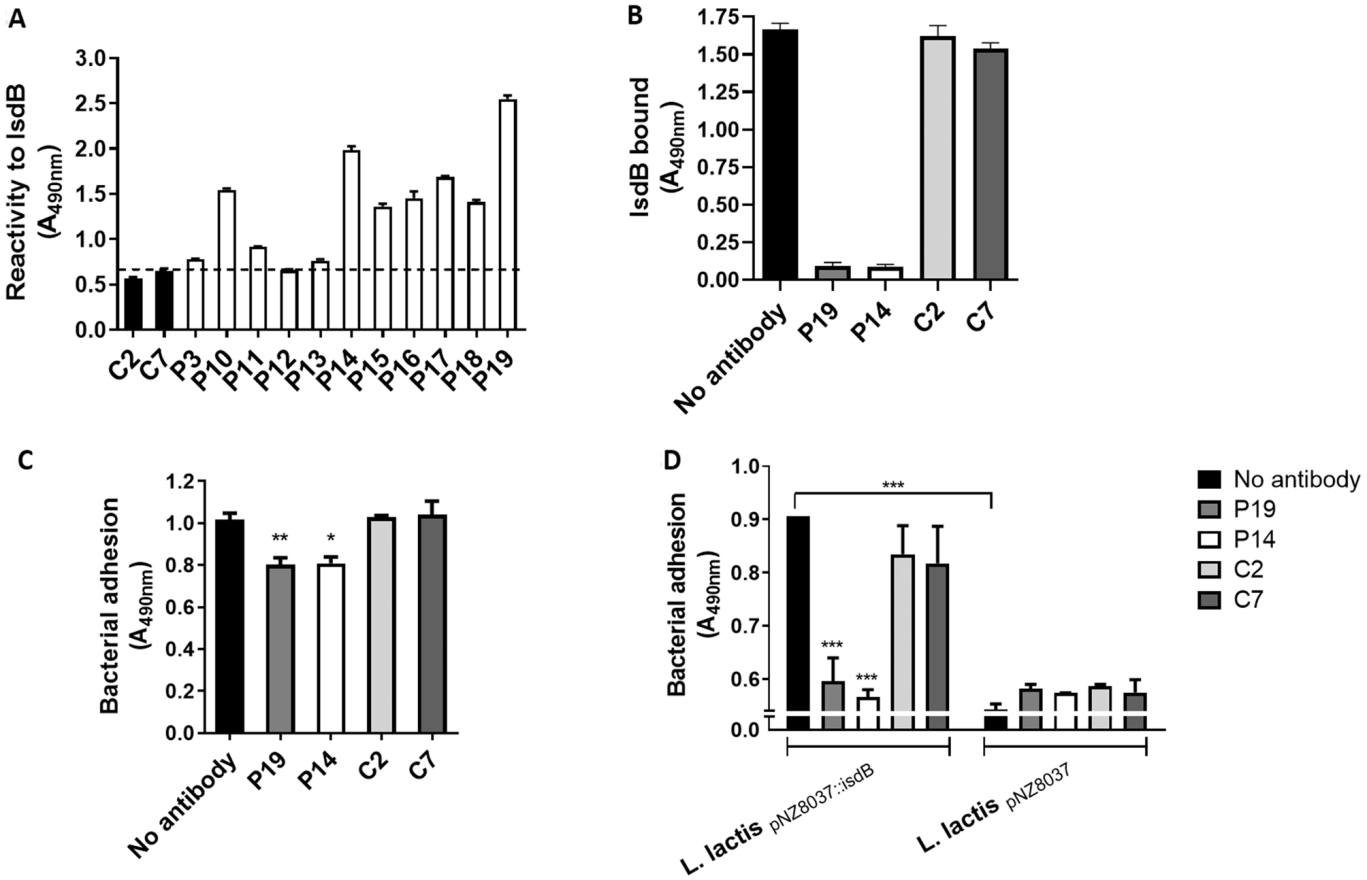
Reactivity of IsdB antibodies from human sera and their effect on IsdB-mediated bacterial adhesion to HUVEC monolayers. **(A)** Patients’ sera reactivity for IsdB protein. Microtiter wells coated with IsdB NEAT1-NEAT2 were probed with IgG isolated from sera of patients with staphylococcal endocarditis. IgG from healthy donors were used as controls. Bound antibody was detected by the addition of rabbit anti-human HRP-conjugated IgG to the wells. Data are expressed as means ± S.D. of triplicate tests. **(B)** Effect of IgG isolated from patients’ sera on IsdB NEAT1-NEAT2 binding to HUVEC monolayers. Confluent HUVEC monolayers were treated with calcium ionophore A23187 and incubated with recombinant IsdB NEAT1–NEAT2 in the presence of the indicated IgG isolated from patients’ sera. Bound IsdB NEAT1–NEAT2 was detected by the addition of a rabbit polyclonal IsdB antibody followed by HRP-conjugated goat anti-rabbit IgG. The binding of IsdB NEAT1–NEAT2 to the monolayers in the presence of control IgG is also reported. Binding observed in the absence of antibodies was set as 100% binding. **(C)** Adhesion of *S. aureus* SH1000 to HUVEC monolayers in the presence of IgG isolated from patients’ sera. HUVEC cell monolayers treated with ionophore A23187 were incubated with cells of *S. aureus* SH1000 WT in the presence of the indicated IgG isolated from patients’ sera or healthy human sera. Adhesion was determined by the addition of an HRP-conjugated rabbit anti-mouse IgG. Bacterial attachment observed in the absence of antibodies was set as 100% adhesion. Statistically significant differences are indicated (*, P < 0.05; **, P < 0.01). **(D)** Adhesion of *L. lactis* ectopically expressing IsdB to HUVEC monolayers in the presence of IgG isolated from patients’ sera. HUVEC cell monolayers treated with ionophore A23187 were incubated with cells of *L. lactis*_pNZ8037_ or *L. lactis*_pNZ8037::isdB_ in the presence of the indicated IgG isolated from patients’ sera or healthy human donors. Adhesion of bacteria to HUVEC monolayers was detected through rabbit anti-*L. lactis* IgG followed by an HRP-conjugated goat anti-rabbit IgG. Statistically significant difference is indicated (***, P < 0.001). Bars reported in **(B–D)** represent means ± SD of triplicate tests.

Considering the role of adhesion to and invasion of endothelia by *S. aureus* and the consequent cardiovascular-associated pathologies such as sepsis and endocarditis, a study was designed where the role of IgG against IsdB on bacterial adhesion to HUVEC cells was investigated. A collection of IgG, previously isolated from patients with *S. aureus* endocarditis^38^, was tested for reactivity to IsdB NEAT1-NEAT2. Although the response varied among the individual IgG preparations, the majority of IgG from patients exhibited a reactivity for IsdB that was higher than that of IgG from sera of healthy donors (Fig. 8A). This observation underlines the *in vivo* relevance of IsdB as an antigen.

Following this, IsdB was pre-incubated with either IgG from the most reactive patients (P14 and P19) or healthy donors (C2 and C7) and then tested for binding to activated HUVEC cells. Patients’ IgG showed a strong blocking effect, while the control IgG affected the binding marginally (Fig. 8B). When SH1000 WT was tested for adhesion to activated HUVEC cells in the presence of IsdB IgG and control IgG, a low but significant blocking effect on adhesion by patients’ IgG was noticed, while no action by the control IgG was recorded (Fig. 8C). The limited inhibitory effect of IsdB IgG can be explained by considering both the action of SpA as a vWF receptor and its activity as an agent capturing the antibodies. Along this line, we investigated the effect of patients' IgG on IsdB-mediated bacterial adhesion to activated HUVEC by *L. lactis*_pNZ8037::*isdB.*_ The *L. lactis* heterologously expressing IsdB exhibited a level of adhesion that was increased by 40% compared to *L. lactis* harboring the empty vector (Fig. 8D). Moreover, pre-incubation of *L. lactis*_pNZ8037::*isdB*_ with patients' IgG almost completely reduced bacterial adhesion to the monolayers, whereas the controls' IgG did not affect the adhesion.

## Discussion

The ability of *S. aureus* to adhere to and colonize the endothelia is strongly associated with the severity of cardiovascular diseases. Several *S. aureus* surface proteins are involved in attachment to endothelial cells or the surface-associated ECM. The fibronectin-binding proteins FnBPA and FnBPB recognize fibronectin in the ECM, and FnBPA/B-mediated adhesion is the prerequisite for endothelial cell invasion by *S. aureus*^39^. Furthermore, ClfA binds to surface-anchored integrin α_V_β_3_ via fibrinogen^40^, and IsdB binds to Vn^26^ and SpA interacts with vWF^7^ in the ECM or with gC1qR on the surface of activated endothelial cells^41^. However, the bacterial and host determinants of endothelial binding have not been fully elucidated. Here we identified vWF as a ligand for *S. aureus* IsdB and provide a comprehensive analysis of the interaction between the bacterial protein and its host binding partner. As reported for Vn, vWF binding to IsdB was strictly related to the conditions required for the optimal expression of the protein (stationary phase of growth and iron-starvation).

Thus, IsdB-expressing bacteria captured soluble vWF and adhered to the immobilized molecule. *L. lactis* ectopically expressing IsdB also attached to surface-coated vWF. The observation that heparin blocked IsdB binding to vWF and the finding that IsdB directly interacted with the A1 domain clearly indicated that the A1 contains the IsdB-binding site. Thus, IsdB and SpA share the same binding site on vWF. It is noteworthy that SpA-mediated adhesion of *S. aureus* to vWF has been demonstrated in conditions of low shear stress^12^, whereas our findings regarding IsdB-binding to vWF were performed in static conditions. However, it cannot be ruled out that IsdB may also play a role in mediating staphylococcal adhesion under shear stress conditions. IsdB binding to vWF significantly increased when the protein was treated with ristocetin, a compound that reproduces *in vivo* high shear stress-induced conformational change of vWF. Thus, it can be concluded that the IsdB binding site is partially hidden in the compact conformation of vWF.

The importance of IsdB-mediated adhesion of *S. aureus* to vWF could be even more relevant *in vivo* considering that the role of SpA as a vWF receptor could be of minor impact in a milieu such as blood where the immunoglobulins could successfully compete with vWF for binding to SpA expressed on the surface of bacteria^7^.

To localize the vWF-binding site(s) on IsdB, the recombinant NEAT1 and NEAT2 modules were tested for their ability to interact with the A1 domain by ELISA. The A1 domain bound dose-dependently to the individual modules and with a binding profile resembling that exhibited using IsdB NEAT1-NEAT2. As determined by SPR, each module interacted with the A1 domain with an affinity comparable to that of IsdB NEAT1-NEAT2. However, the NEAT2 module showed an affinity for A1 that was higher than that of NEAT1 that is consistent with the low sequence identity and the different functionality of the modules.

The ability of A1 to bind both the modules is reminiscent of Vn binding to each NEAT domain. However, Vn did not compete with the binding of A1 to IsdB (data not shown), indicating that A1 and Vn recognize and bind to different subsites within the IsdB domains and, possibly, with different binding mechanisms. The evidence that high ionic strength significantly reduced the IsdB interaction with the A1 domain, as well as molecular docking analysis, provide clues that electrostatic bonds could be involved in the vWF-IsdB interaction.

We also demonstrated that IsdB mediates *S. aureus* adhesion to vWF on activated HUVEC cells. Furthermore, we show that adhesion directly involves the A1 domain as indicated by the specific inhibitory effect of the A1 mAb named 6D1 on the process. Thus, a scenario can be envisaged where activated endothelial cells secrete ultra large vWF fibers that run parallel to the direction of flow in the blood^42^. vWF multimers can also rebind to the endothelial surface via integrin α_v_β_3_^42^ or, in case of endothelial damage or inflammation, to subendothelial matrix molecules such as fibrin monomers^43^, collagens^44–46^ or fibronectin^47^. Due to the effect of shear stress, the globular, concealed A1 domain in vWF fibers becomes exposed and allowed to interact with *S. aureus* via specifically up-regulated IsdB in the iron-limited conditions of the blood. These events allow the pathogen to withstand the strong current of the blood flow and create the prerequisite of vascular infections such as infective endocarditis. In support of the crucial role of A1 in bacterial adherence, in the presence of heparin, a binder of A1, both fiber formation and bacterial adhesion are simultaneously reduced^5^.

The importance of bacterial receptor/vWF interaction in vascular infections is underlined by the consideration that, among the staphylococci, only *S. aureus*^7,8,34^ and the coagulase-negative *Staphylococcus lugdunensis* are able to bind vWF and are more effective in causing endocarditis compared to other staphylococcal species^48^. The nature of the vWF receptor in *S. lugdunensis* remains elusive, despite its potential importance in the pathogenesis associated with this bacterium^49,50^. Perhaps the IsdB orthologue of *S. lugdunensis* binds vWF and promotes endothelial colonization and invasion.

The functional aspects of *S. aureus* binding to vWF can be correlated with the pathophysiological consequences of this interaction such as infective endocarditis, sepsis, and cardiovascular complications. Infection of the heart valves is triggered by the attachment of circulating bacteria to the endocardium and the formation of bacterial vegetations, which are embedded in fibrin and platelets. Bacterial growth occurs within cells and the matrix inside vegetations, making it difficult for the host immune system to control or eradicate the ongoing infection. Therefore, in the perspective of future therapeutic interventions, the acquisition of information on the immune response by the host remains essential.

Along this line, we examined the reactivity of IgG isolated from patients with *S. aureus* endocarditis and their possible interference with the vWF/IsdB interaction. A considerable proportion of isolated IgG from a collection of human sera showed a significant reactivity to IsdB. Moreover, anti-IsdB antibodies blocked the binding of recombinant IsdB to activated endothelial cells and interfered with the adhesion of *L. lactis* ectopically expressing IsdB to HUVEC cells. On the other hand, the antibodies affected less, although significantly, *S. aureus* adhesion to endothelial cells. The reduced efficacy of immune IgG on adhesion of SH1000 to HUVEC cells may be attributable to the action of SpA as a vWF receptor per se and/or to its IgG neutralizing activity. Moreover, the involvement of other staphylococcal vWF-binding partners in the process cannot be excluded. Finally, although our data suggest a role for IsdB as a host colonization/invasion factor and as a potential player in *S. aureus* pathogenesis, it remains to be determined whether IsdB can be used as potential vaccine component. Indeed, it has been found that a vaccine including IsdB induced a higher mortality rate than the placebo in human patients^51,52^. Thus, additional studies are needed to better estimate the pathophysiological role of IsdB and its therapeutic value.

## Methods

### Bacterial strains and culture conditions

All strains used in this study are listed in Table 1. *S. aureus* cells^26,53,54^ were grown overnight in brain heart infusion (BHI) (VWR International Srl, Milan, Italy) or RPMI 1640 (Sigma-Aldrich, St. Louis, MO, USA) medium at 37 °C with shaking. *L. lactis* cells carrying the expression vector alone (pNZ8037)^55^ or harboring the *isdB* gene (pNZ8037::*isdB*)^25^ were grown overnight in BHI medium supplemented with chloramphenicol (10 μg/ml) at 30 °C without shaking. Cultures of *L. lactis* were diluted at 1:100 in the same medium and allowed to reach the exponential phase of growth. Nisin (6.4 ng/ml) was added, and cultures were allowed to grow overnight as above. In those experiments where a defined number of cells were used, bacteria were harvested from the cultures by centrifugation, washed, and suspended in phosphate-buffered saline (PBS) at OD_600nm_ = 1.0. *Escherichia coli* BL21 (DE3) (Invitrogen, Carlsbad, CA, USA) transformed with vector pQE30 or pET28a (Integrated DNA Technologies, Leuven, Belgium) was grown in Luria agar and Luria broth (VWR International Srl) containing 100 μg/ml ampicillin or kanamycin, respectively, at 37 °C with shaking.

**Table 1:** Abbreviations used are as follows: Cm^r^, chloramphenicol resistance, Tc^r^, tetracycline resistance.

Bacterial strain	Relevant properties	Reference
***S. aureus***		
SH1000 WT	Laboratory strain. *rsbU* ^+^ derivative of *S. aureus* 8325‐4	^53^
SH1000 *spa*	*spa*::Tc^r^ transduced from 8325–4 *spa::Tc*^*r*^	^54^
SH1000 *isdB*	*isdB gene deleted by allelic exchange*	^26^
***L. lactis***		
NZ9800 (pNZ8037)	*Expression vector with a nisin-inducible promoter, Cm*^*r*^	^25^
NZ9800 (pNZ8037::*isdB*)	*isdB gene cloned in pNZ8037 Cm*^*r*^	^26^
***E. coli***		
XL1-Blue	*E. coli* cloning host	Stratagene
BL21 (DE3)	*E. coli* cloning host	Invitrogen

### Plasmid and DNA manipulation

Plasmid DNA (Table 2) was isolated using the NucleoSpin Plasmid kit (Macherey–Nagel GmbH & Co. KG, Düren, Germany), according to the manufacturer’s instructions, and transformed into *E. coli* XL1-Blue or BL21 (DE3) cells using standard procedures^56^. Transformants were screened by restriction analysis and verified by DNA sequencing (Eurofins Genomics, Milan, Italy). Cloning of IsdB NEAT1-NEAT2 (aa residues 48–480) was performed as reported by Miajlovic et al.^57^. Cloning of IsdB NEAT1 (aa residues 144–270) and IsdB NEAT2 (aa residues 334–458) domains was performed as reported previously^26^.

**Table 2:** Abbreviations used as follows: Amp^R^, ampicillin resistance; Kan^R^, kanamycin resistance.

Plasmid	Feature	Marker	Reference
pQE30	*E. coli* vector for the expression of hexa-His-tagged recombinant proteins	Amp^R^	Qiagen
pQE30::*isdB* NEAT1-NEAT2	pQE30 encoding the NEAT1-NEAT2 domains of IsdB protein from *S. aureus* SH1000	Amp^R^	^57^
pQE30::*isdB*-NEAT1	pQE30 derivative encoding the NEAT1 domain of IsdB protein from *S. aureus* SH1000	Amp^R^	^26^
pQE30::*isdB* NEAT2	pQE30 derivative encoding the NEAT2 domain of IsdB protein from *S. aureus* SH1000	Amp^R^	^26^
pET28a	*E. coli* vector for the expression of hexa-His-tagged recombinant proteins	Kan^R^	Millipore Sigma
pET28a::*vWF-A1*	pET28a derivative encoding the A1 domain of the human von Willebrand factor	Kan^R^	This study

The synthetic gene fragment corresponding to the A1 domain (aa residues 460–730) of human vWF, modified based on the protocol of Chudapongse et al*.*^58^, was purchased by Eurofins Genomics. Cloning of the A1 domain was performed following the NEBuilder® HiFi DNA Assembly according to the manufacturer’s instructions (New England Biolabs, MA, USA). The primers used to amplify the A1 domain and the pET28a vector (Supplementary Table S1) were purchased from Integrated DNA Technologies.

### Expression and purification of recombinant proteins

Recombinant A1 domain was expressed from pET28a (Millipore-Sigma, MA, USA) in *E. coli* BL21 (DE3) (Invitrogen). Recombinant proteins IsdB NEAT1–NEAT2, IsdB NEAT1, and IsdB NEAT2 were expressed as previously reported^26^. Overnight starter cultures were diluted at 1:40 in Luria broth containing the appropriate antibiotics (see above) and incubated with shaking until the culture reached the exponential phase (A_600_ = 0.4–0.6). Recombinant protein expression was induced by the addition of 1 mM (final concentration) isopropyl 1-thio-β-D-galactopyranoside (IPTG) (Inalco, Milan, Italy) to the culture. After 4 h, bacterial cells were harvested by centrifugation and frozen at -80 °C. Cells were re-suspended in lysis buffer (50 mM NaH_2_PO_4_, 300 mM NaCl, pH 8.0) containing 1 mM phenyl-methane-sulfonyl-fluoride (PMSF) (Sigma-Aldrich) and 20 μg/mL DNase (Sigma-Aldrich) and lysed by sonication (70% amplitude, 12 × 30″ on/off, 1′30″ interval between sonication steps). The cell debris was removed by centrifugation and proteins purified from the supernatants by Ni^+2^-affinity chromatography on a HiTrap chelating column (GE Healthcare, Buckinghamshire, UK). Protein purity was assessed by 10% SDS-PAGE and Coomassie Brilliant Blue staining. A bicinchoninic acid protein assay (Pierce, Rockford, IL, USA) was used to measure the concentration of purified proteins.

Recombinant proteins FnBPA_194-511_^59^, FnBPB_163-480_^60^, IsdA_47-313_^21^, IsdH_41-660_^23^, ClfA_221-559_^61^, ClfB_201-542_^62^, CNA_31-344_^63^, SdrE_53-606_^64^, SdrD_36-568_^64^ were all expressed with His_6_ N-terminal affinity tags and purified as reported above.

### Reagents, proteins, and antibodies

BSA (bovine serum albumin), protease-free DNase I, skim milk, von Willebrand factor, heparin, chondroitin sulphate, heparan sulphate, lysostaphin, nisin, protein A (SpA_37-485_), biotin, avidin-HRP, trypsin, calcium ionophore A23187 were purchased from Sigma-Aldrich. Collagens type I, type III, type IV, and type VI were purchased by Merck (Darmstadt, Germany). Ristocetin was from Hyphen BioMed (Neuville-sur-Oise, France). IgG were isolated from patients’ with infective endocarditis as previously reported^38^, all methods were carried out in accordance with relevant guidelines and regulations, all experimental protocols were approved by the ethical board of the University of Pavia and informed consent was obtained from all human participants. Anti-A1 6D1 monoclonal antibody was raised as previously described^34^. αThrombin and PPACK were bought from Haematologic Technologies (Essex Junction, VT, USA). Sensor Chip NTA and Ni^2+^ Sepharose 6 Fast Flow resin were purchased by Cytiva Lifesciences (Washington, USA). IsdB antibody was raised in a rabbit by routine immunization procedure using purified IsdB NEAT1-NEAT2 as the antigen. Anti-*L. lactis* antibody was raised in a rabbit by routine immunization procedure using heat-inactivated *L. lactis* NZ9800 cells as the antigen. Rabbit anti-human von Willebrand factor IgG and rabbit anti-mouse or goat anti-rabbit horseradish peroxidase (HRP)-conjugated secondary antibodies were purchased from Dako Cytomation (Glostrup, Denmark). Monoclonal mouse HRP-conjugated α-polyHistidine antibody was purchased from Abcam (Cambridge, UK).

### Release of CWA proteins from *S. aureus* and detection of vWF-binding activity

*Lysostaphin digestion.* CWA proteins from *S. aureus* were released by following the protocol described by Pietrocola *et al*^26^. Briefly, bacteria were grown to the stationary phase, either in RPMI or BHI medium, harvested by centrifugation at 7000 × *g* for 15 min at 4 °C, washed three times with PBS, and resuspended to an A_600_ = 2.0 in lysis buffer (50 mM Tris–HCl, 20 mM MgCl_2_, pH 7.5) supplemented with 30% (w/v) raffinose. Cell wall proteins were solubilized from *S. aureus* by incubation with lysostaphin (200 μg/ml) for 20 min at 37 °C in the presence of protease inhibitors (Complete Mini; Sigma-Aldrich). Protoplasts were recovered by centrifugation at 6000 × *g* for 20 min, while the supernatant taken as the wall fraction was concentrated by treatment with 20% (v/v) trichloroacetic acid (TCA) for 30 min at 4 °C. The precipitated proteins were washed twice with ice-cold acetone and dried overnight.

#### SDS-PAGE and Western blotting

Proteins released by lysostaphin digestion were boiled for 10 min in sample buffer (0.125 M Tris–HCl, 4% (w/v) SDS, 20% (v/v) glycerol, 10% (v/v) β-mercaptoethanol, 0.002% (w/v) bromophenol blue), separated by 10% (w/v) SDS-PAGE and electroblotted onto a nitrocellulose membrane (GE Healthcare). After blocking with 5% (w/v) skim milk (Sigma-Aldrich) in PBS overnight at 4 °C, the membrane was probed with either 2 μg/ml of human vWF in PBS for 1 h at 22 °C followed by rabbit polyclonal anti-vWF IgG (1:5000) and HRP-conjugated goat anti-rabbit IgG (1:10,000) in 1% (w/v) skim milk or with rabbit polyclonal anti-IsdB IgG (1:5000) and HRP-conjugated goat anti-rabbit IgG (1:10,000) in 1% (w/v) skim milk. Blots were developed using the ECL Advance Western blotting detection kit (GE Healthcare), and images of the bands were captured by an ImageQuantTM LAS 4000 mini-biomolecular imager (GE Healthcare).

### ELISA-type solid-phase binding assays

#### Binding of vWF to IsdB and blocking experiments

The ability of soluble vWF to bind to immobilized recombinant CWA proteins (SpA_37-485_, FnBPA_194-511_, FnBPB_163-480_, IsdA_47-313_, IsdB_48–480_, IsdH_41-660_, ClfA_221-559_, ClfB_201-542_, CNA_31-344_, SdrE_53-606_, SdrD_36-568_) was determined by an ELISA assay. Microtiter wells were coated overnight at 4 °C with 1 μg/well of each protein in 0.1 M sodium carbonate, pH 9.5. The plates were washed three times with 0.5% (v/v) Tween 20 in PBS (PBST). To block additional protein binding sites, the wells were treated for 1 h at 22 °C with 2% (v/v) BSA in PBS and incubated for 1 h at 22 °C with 1 μg/well of vWF in PBS. The plates were incubated with a rabbit polyclonal anti-human vWF IgG (1:1000 in 1% (v/v) BSA) and incubated for 1 h at 22 °C. The wells were then incubated with an HRP-conjugated goat anti-rabbit IgG (1:1000 in 1% (v/v) BSA) for 45 min at 22 °C. After washing, *o*-phenylenediamine dihydrochloride was added to the wells and the absorbance at 490 nm was determined using an ELISA plate reader (BioRad, Hercules, CA, USA).

To determine the effect of vWF ligands (heparin, and different types of collagens) on the IsdB-vWF interaction, microtiter wells, coated with 1 μg of vWF, were incubated with 1 μg of IsdB in the presence or absence of 10 μg/well of each ligand. IsdB binding to vWF was detected by the addition of a rabbit polyclonal anti-IsdB IgG (1:2000) followed by HRP-conjugated goat anti-rabbit IgG (1:1000).

The binding of 1 μg of IsdB to surface coated vWF (1 μg/well) in presence of increasing concentrations (from 0 to 10 μg/well) of heparin, chondroitin sulfate, or heparan sulfate was detected with IsdB antibody as reported above.

To assess the effect of ionic strength on the IsdB-vWF interaction, microtiter wells coated with 1 μg of vWF were incubated with 1 μg of IsdB in presence of increasing concentrations of NaCl (from 0 to 1 M). Complex formation was detected by incubation of the wells with the IsdB antibody.

The dose-dependent binding of IsdB to surface-coated human vWF or A1 domain (1 μg/well) was evaluated by incubation of the plates with increasing concentrations of IsdB (from 0 to 5 μg/well). Bound protein was revealed as reported above.

Binding of IsdB to the vWF A1 domain was performed incubating immobilized A1 domain (1 μγ/well) with IsdB (1 μg/well) in the presence of 250 ng of 6D1 or anti-ClfA monoclonal antibodies and detected as reported above.

Binding of increasing concentrations of the A1 domain (from 0 to 5 μg/well) to immobilized IsdB NEAT1–NEAT2 or its subregions NEAT1 and NEAT2 (1 μg/well) was also assessed and detected by the addition of anti-human vWF IgG.

To assess the allosteric conformation change of vWF and the A1 domain exposure by ristocetin treatment, vWF (10 μg /ml) was treated with 0.5 mg/ml of ristocetin and the complex used to coat microtiter wells (100 μl). The A1 domain exposure was evaluated by the addition of the anti-A1 domain monoclonal antibody 6D1 (250 ng/well) followed by HRP-conjugated rabbit anti-mouse IgG (1:1000) to the wells.

The impact of the conformational change of the ristocetin-treated vWF was evaluated by measuring the binding of soluble vWF (1 μg/well) to immobilized IsdB (1 μg/well). The complex formation was detected using anti-human vWF IgG as previously reported.

#### Bacterial adhesion to surface-coated vWF

The ability of *S. aureus* or *L. lactis* ectopically expressing IsdB to adhere to surface-coated vWF was evaluated by ELISA-based assay. Microtiter wells coated with vWF were incubated with cells (A_600_ = 1.0) of *S. aureus* SH1000 and its *isdB* mutant obtained from cultures grown to stationary phase in RPMI and suspended in 0.5% (v/v) BSA. The wells were extensively washed with PBST, blocked with 2% (v/v) BSA, and incubated with 100 µl bacterial suspensions for 1 h at 22 °C. The expression of SpA on cell surface was exploited to detect bacteria adhesion by incubating the plates for 45 min with HRP-conjugated rabbit anti-mouse IgG (1:1000).

Binding of HRP-conjugated rabbit anti-mouse IgG (1:1000) to surface-coated *S. aureus* SH1000 and its *isdB* isogenic mutant cells (1 × 10^7^) was measured in a standard ELISA assay.

Binding of non-immune IgG such as HRP-conjugated goat anti-rabbit IgG or anti-*L. lactis* IgG to IsdB was determined by incubating surface-coated recombinant IsdB (1 μγ/well) with the antibodies and antibody binding was detected as reported above.

Adhesion of *L. lactis* to vWF was performed by incubating plates coated with vWF with cells of *L. lactis* expressing IsdB (*L. lactis*_pNZ8037::*isdB*_*)* and the strain carrying the empty vector (*L.lactis*_pNZ8037_) obtained from cultures grown in BHI. *L. lactis* binding to surface-coated vWF was detected by incubating for 1 h at 22 °C with rabbit polyclonal anti-*L. lactis* IgG (1 µg/well) followed by an HRP-conjugated goat anti-rabbit IgG (1:1000).

#### Reactivity of IgG from patients with infective endocarditis against IsdB

To test the reactivity of IgG from the collection of infective endocarditis sera IsdB NEAT1-NEAT2 was immobilized onto microtiter wells (1 µg/well). After blocking with BSA, the wells were incubated with antibodies (1 μg/well) from patients and healthy donors. The binding of antibodies was revealed by the addition of a polyclonal rabbit anti‐human IgG (1:1000).

### Capture of vWF by *S. aureus* cells

Cells of *S. aureus* strain SH1000 WT or the *isdB* mutant, grown to stationary phase in RPMI, were harvested by centrifugation at 7000 × *g* at 4 °C for 15 min, washed three times with PBS, and resuspended to an A_600_ = 1.0 in PBS. Cells were then incubated with human vWF (5 µg/mL) for 1 h at 22 °C under constant shaking. The extraction of vWF captured by bacteria was conducted as previously described^22^. Briefly, bacteria were treated with the extraction buffer (125 mM Tris–HCl, pH 7.0, 2% (w/v) SDS) for 3 min at 95 °C and finally centrifuged at 10.000 × *g* for 3 min. The supernatants were subjected to 5% (w/v) SDS-PAGE under reducing conditions, and the proteins were electrotransferred to a nitrocellulose membrane. The membrane was incubated with a rabbit polyclonal vWF antibody followed by HRP-conjugated goat anti-rabbit IgG. The band intensities were quantified with Quantity One software (Bio-Rad).

### Surface plasmon resonance

For performing SPR measurements on a NTA-Ni^2+^ sensor chip (see below), the recombinant 6xHis-tag-A1 was treated with α-thrombin to remove the 6xHis tag to yield vWF A1 with an additional 17-amino acid segment at the N-terminus. The fused protein (1 mg/ml, 0.5 ml) was treated at an enzyme:substrate molar ratio of 1:200 in PBS, for 1 h at 25 °C. The reaction was quenched by adding 1 μM (D)-Phe-Pip-Arg-chloromethylketone, as an irreversible thrombin inhibitor, while A1 was purified by the batch mode procedure. Briefly, the reaction mixture was incubated with 25 μl of Ni^2+^-Sepharose-6 Fast Flow resin at 22 °C for 1 h under gentle stirring on an orbital shaker. The supernatant, containing the purified A1, was then collected and protein purity and chemical identity assessed by non-reducing 4–12% (w/v) SDS-PAGE, high-resolution mass spectrometry and by Dot Blot analysis (not shown), using anti-His tag IgG as a probe, all confirming the removal of the 6xHis-tag.

SPR analyses were carried out on a dual flow-cell Biacore X-100 instrument (Cytiva, Uppsala, Sweden) as described^65^. 6xHis-tag-IsdB proteins (i.e. the ligands) were non-covalently immobilized onto a Ni^2+^-chelated nitrilotriacetate (NTA) carboxymethyldestrane sensor chip and incremental concentrations of A1 domain (i.e. the analyte) lacking the 6xHis-tag were loaded at incremental concentrations in the mobile phase, following the single-cycle operation mode. The Ni^2+^-NTA/6xHis-IsdB chip assembly was prepared as follows: the NTA chip (Cytiva) was first washed (flow-rate: 30 μl/min) with 0.35 M EDTA, pH 8.3 (contact time: 60 s) and then loaded with 0.5 mM NiCl_2_ (120 μl, contact time: 240 s); excess Ni^2+^ was removed by injecting 3 mM EDTA (contact time: 180 s); finally, a solution of 6xHis-IsdB (130 μl, 2 μg/ml) was injected on the sensor chip (contact time: 180 s) to yield a final immobilization level of 655 response units (RU). Next, the Ni^2+^-NTA/6xHis-IsdB chip was challenged (flow-rate: 30 μl/min; contact time: 120 s) with increasing concentrations of vWF A1. All measurements were carried out at 25 °C in HBS-EP + buffer (10 mM HEPES, pH 7.4, 0.15 M NaCl, 50 μM EDTA, 0.005% v/v polyoxyethylene sorbitan). After each set of measurements, the NTA chip was regenerated by a pulse of regeneration buffer (350 mM EDTA). Each sensogram was subtracted for the corresponding baseline, obtained on the reference flow cell and accounting for nonspecific binding, i.e. typically less than 2% of RU_max_. The binding data were analyzed using the BIAevaluation software vs 2.0. The sensorgrams (Fig. 6, black curves) were fitted with theoretical curves obtained by simulations based on several different 1:1 stoichiometric binding models: (i) simple analyte-ligand interaction, (ii) bivalent analyte, and iii) heterogeneous ligand binding model^35,36^. The best fit, as evaluated from the χ^2^ values of experimental and simulated sensorgrams, was obtained using the heterogeneous ligand binding model, which assumes the existence of two differently populated orientations (i.e., L1 and L2) of the immobilized ligand, allowing the exposure of different ligand surfaces which are variably accessible for interaction with the analyte and bind the analyte (i.e. A1 domain) with different affinities (i.e. K_D1_ and K_D2_). This is even more likely to occur with highly charged IsdB proteins bound on the NTA sensor chip, formed by the negatively charged chelating agent NTA and the positive Ni^2+^ ions. The relative abundance of L_1_ and L_2_ were estimated from their RU_max_ values, obtained as a fitting parameter, e.g. L_1_ = [RU_max1_/(RU_max1_ + RU_max2_)] × 100^35,36^.

### Computational methods

Electrostatic potential calculations were carried out using the APBS program^66,67^, run on the crystallographic structure of IsdB_E chain (5vmm.pdb)^18^, after removal of Hb coordinates, and on the crystallographic structure of the A1 domain bound to the platelet thrombin receptor GpIbα (1u0n.pdb)^68^, after removal of the receptor coordinates. Calculations were performed using a solvent dielectric of 78.14 and a protein dielectric of 2.0 at 310 K in 150 mM NaCl.

### Binding and adhesion assays to endothelial cells

#### IsdB binding to endothelial cells

Human umbilical vein endothelial cells (HUVEC) from a single donor (Lonza, Spain) were kindly provided by the Researchers of the Neonatal Unit and Neonatal Intensive Care Unit, Fondazione IRCCS Policlinico S. Matteo, Pavia, Italy and cultured as previously reported^40^. To examine the binding of recombinant IsdB NEAT1-NEAT2 to endothelial cells, HUVECs were cultured onto 96-microtiter wells. Monolayers (8 × 10^4^ cells/well) were treated with 0.1 mM calcium ionophore A23187 (Sigma-Aldrich) for 10 min at 22 °C, washed three times with PBS, and then fixed with 3% (w/v) paraformaldehyde in PBS for 10 min. The wells were thoroughly rinsed with PBS, blocked with BSA (v/v) 2% in PBS for 1 h, and then incubated with increasing concentrations of recombinant IsdB (0.63–2.5 µg/well) in PBS for 1 h. After extensive washing, IsdB binding to the wells was detected by addition to the wells of rabbit polyclonal IsdB IgG followed by HRP-conjugated goat anti-rabbit IgG.

#### Bacterial adherence to endothelial cells

The ability of *S. aureus* cells to adhere to HUVEC cells was assessed by an ELISA-based assay. 100 μl of bacterial suspensions (A_600_ = 1.0) of *S. aureus* strain SH1000 WT and its isogenic *isdB* mutant obtained from cultures grown in RPMI were added to ionophore-treated HUVEC monolayers and the wells incubated for 1 h. The attached bacteria were detected by incubating the wells with HRP-conjugated rabbit anti-mouse IgG (1:1000) for 45 min at 22 °C. To test the effect of the anti-A1 monoclonal antibody (mAb) 6D1 on the adhesion of *S. aureus* SH1000 to HUVEC monolayers, the assay was performed in the presence of 250 ng/well of the 6D1 or isotype matched anti-ClfA mAb and bacterial attachment determined as above.

#### Inhibitory activity of patients’ IgG on the interaction of IsdB with vWF expressed on endothelial cells

The ability of the patients’ IgG to interfere with the binding of recombinant IsdB NEAT1-NEAT2 to ionophore-treated and fixed endothelial cells was determined by incubating the wells with 2.5 μg IsdB in the presence of the indicated IgG (10 μg/well) for 1 h at 22 °C. The binding of IsdB to the cells was detected as reported above. To analyse the effect of patients’ IgG on adherence of staphylococci to ionophore-treated HUVEC cells, 100 μl of a *S. aureus* SH1000 WT suspension (A_600_ = 1.0) and 10 μg/well of the indicated IgG were simultaneously added to the monolayers and the wells incubated for 1 h at 22 °C. Bacterial adherence was detected by incubating the wells with an HRP-conjugated rabbit anti-mouse IgG (1:1000). A similar adhesion protocol was used to test the effect of patients’ IgG on adhesion *of L. lactis*_*pNZ8037::isdB*_ and *L. lactis*_*pNZ8037*_ to the monolayers. *L. lactis* adherence to the cells was determined by adding to the wells rabbit anti-*L. lactis* IgG (1 µg/well) followed by an HRP-conjugated goat anti-rabbit IgG (1:1000).

### Statistical methods

Analyses were performed using Prism 4.0 (GraphPad). Comparison of more than two groups were performed with the one-way ANOVA followed by Bonferroni’s post hoc tests. The two-tailed Student's t-test was employed to compare two groups. *P* values < 0.05 were considered statistically significant (*,* P* < 0.05, **, *P* < 0.01, ***, *P* < 0.001).

## Supplementary Information

Supplementary Information.

Supplementary Figure S3.
