# Supplementary material for: Staphylococcus aureus iron-regulated surface determinant B (IsdB) protein interacts with von Willebrand factor and promotes adherence to endothelial cells
Source: Sci Rep. 2021 Nov 23;11:22799. doi: 10.1038/s41598-021-02065-w (PMC8611056; doi:10.1038/s41598-021-02065-w)
Supplement: Supplementary file 1 — Supplementary Information. [file 41598_2021_2065_MOESM1_ESM.docx]

***Staphylococcus aureus* iron-regulated surface determinant B (IsdB) protein interacts with von Willebrand factor and promotes adherence to endothelial cells**

**Mariangela J Alfeo^1^, Anna Pagotto^2^, Giulia Barbieri^3^, Timothy J Foster^4^, Karen Vanhoorelbeke^5^, Vincenzo De Filippis^2^, Pietro Speziale^1^ & Giampiero Pietrocola^1*^**

^1^Department of Molecular Medicine, Biochemistry Unit, Viale Taramelli 3/b, 27100 Pavia, Italy.

^2^Department of Pharmaceutical and Pharmacological Sciences, Padua University School of Medicine, Via Marzolo 2, 35100 Padua, Italy.

^3^Department of Biology and Biotechnology "Lazzaro Spallanzani", University of Pavia, Pavia, Italy.

^4^Microbiology Department, Trinity College Dublin, Dublin, Ireland.

^5^Laboratory for Thrombosis Research, KU Leuven Campus Kulak Kortrijk, Kortrijk, Belgium.

*email: [giampiero.pietrocola@unipv.it](mailto:giampiero.pietrocola@unipv.it)

**SUPPLEMENTARY MATERIAL 1**

**Fig. 1S – Binding of IsdB NEAT1-NEAT2 to ristocetin-treated vWF**. A, binding of the monoclonal antibody 6D1 to vWF treated with ristocetin (0.5 mg/ml) for 30 min at 37 °C. Ristocetin-treated vWF was immobilized onto microtiter wells and incubated with the mAb 6D1. The binding of the antibody was detected by the addition of an HRP-conjugated rabbit anti-mouse IgG. The binding of 6D1 to untreated vWF is reported as a control. A statistically significant difference is indicated (*, *P* < 0.05). B, binding of ristocetin-treated vWF to immobilized IsdB NEAT1-NEAT2. Surface-coated IsdB NEAT1-NEAT2 was incubated with ristocetin-treated vWF. The binding of the ligand to immobilized protein was detected by the addition of a rabbit polyclonal vWF antibody followed by HRP-conjugated goat anti-rabbit IgG.A statistically significant difference is indicated (*, *P* < 0.05). Data are expressed as means ± SD of triplicate tests.

 **2SA 2SB**

**Fig. 2S – Role of electrostatic forces on the interaction of IsdB with vWF.** A, the effect of ionic strength on the binding of IsdB NEAT1-NEAT2 to vWF. The binding of IsdB NEAT1-NEAT2 to immobilized vWF was tested under increasing concentrations of NaCl. Binding of the ligand was detected by addition to the wells of a rabbit anti-IsdB IgG, as reported in Fig.7. Error bars show S.D. of the means from three independent determinations, each performed in triplicate. B, Surface electrostatic properties of vWF-A1 and IsdB. Ribbon drawings and surface electrostatic potential of IsdB (5vmm.pdb) (*a,b,c,d*) and A1 (1u0n.pdb) (*e,f,g,h*). The protein surfaces are color-coded according to their electrostatic potential, from −1 kcal/mol/e− (deep red) to +1 kcal/mol/e− (deep blue). Electrostatic potential calculations were carried out using the APBS program.

**3SA 3SB**

Fig. 3S. A, HRP-conjugated antibody binding to *S. aureus* SH1000 WT and the *isdB* mutant. Bacteria (1 x 10^7^) were immobilized onto microtiter wells, tested for binding of HRP-conjugated rabbit anti-mouse IgG and the antibody binding detected as reported in Methods. B, Reactivity of recombinant IsdB with non-immune IgG. Recombinant IsdB was immobilized onto microtiter wells and then incubated with HRP-conjugated goat anti-rabbit IgG. The binding to IsdB of rabbit anti-IsdB followed by HRP-conjugated goat anti-rabbit IgG is also reported as a control. Alternatively, the wells were incubated with rabbit anti- *L. lactis* IgG followed by addition of HRP-conjugated goat anti-rabbit IgG. The binding of secondary antibodies was detected as reported in Methods. Data are expressed as means ± SD of triplicate tests.

**4S**

***

**Fig. 4S. Effect of mAb 6D1 on the binding of IsdB to A1.** Recombinant A1 domain was immobilized onto microtiter wells and incubated with IsdB NEAT1-NEAT2 in the presence of mAb 6D1 or an isotype control mAb. Complex formation between IsdB and A1 was detected by addition to the wells of a rabbit anti-IsdB IgG followed by HRP-conjugated goat anti-rabbit IgG. Statistically significant difference is indicated (***, *P* < 0.001). Data are expressed as means ± SD of triplicate tests.

**Table S1. Primers used in this study**

| Primer | Sequence (5’-3’) | 5’ restriction site |
| --- | --- | --- |
| A1 (460-730) F | agcaaatgggtcgcggatccgatgttgtcaacctcacctctgaagc | BamHI |
| A1 (460-730) R | tgtcgacggagctcgaattcTCAcaagagccccgggcc | EcoRI |
| pET28a-A1 F | gaattcgagctccgtcgac | EcoRI |
| pET28a-A1 R | ggatccgcgacccatttg | BamHI |
