# Supplementary material for: Staphylococcus aureus iron-regulated surface determinant B (IsdB) protein interacts with von Willebrand factor and promotes adherence to endothelial cells
Source: Sci Rep. 2021 Nov 23;11:22799. doi: 10.1038/s41598-021-02065-w (PMC8611056; doi:10.1038/s41598-021-02065-w)
Supplement: Supplementary file 2 — Supplementary Figure S3. [file 41598_2021_2065_MOESM2_ESM.pdf]

Staphylococcus aureus iron-regulated surface determinant B (IsdB) protein interacts with von Willebrand factor and promotes adherence to endothelial cells

Mariangela J Alfeo[1], Anna Pagotto[2], Giulia Barbieri[3], Timothy J Foster[4], Karen Vanhoorelbeke[5], Vincenzo De Filippis[2], Pietro Speziale[1] & Giampiero Pietrocola[1*]

[1]Department of Molecular Medicine, Biochemistry Unit, Viale Taramelli 3/b, 27100 Pavia, Italy.

[2]Department of Pharmaceutical and Pharmacological Sciences, Padua University School of Medicine, Via Marzolo 2, 35100 Padua, Italy.

[3]Department of Biology and Biotechnology "Lazzaro Spallanzani", University of Pavia, Pavia, Italy.

[4]Microbiology Department, Trinity College Dublin, Dublin, Ireland.

[5]Laboratory for Thrombosis Research, KU Leuven Campus Kulak Kortrijk, Kortrijk, Belgium.

*email: giampiero.pietrocola@unipv.it

SUPPLEMENTARY MATERIAL 2

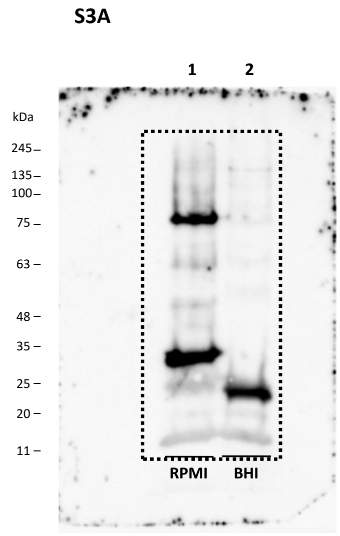

1: *S. aureus* SH1000 *spa* in RPMI
2: *S. aureus* SH1000 *spa* in BHI

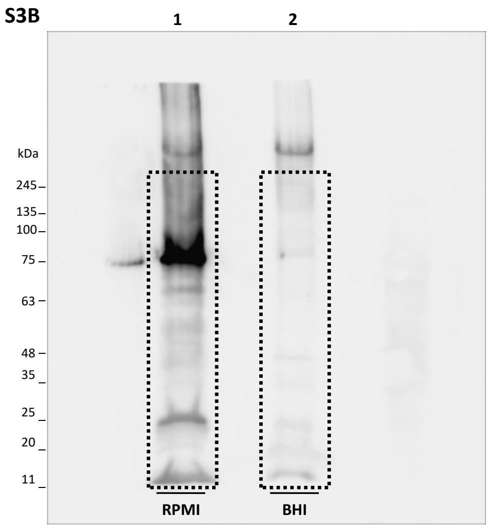

1: *S. aureus* SH1000 *spa* in RPMI
2: *S. aureus* SH1000 *spa* in BHI

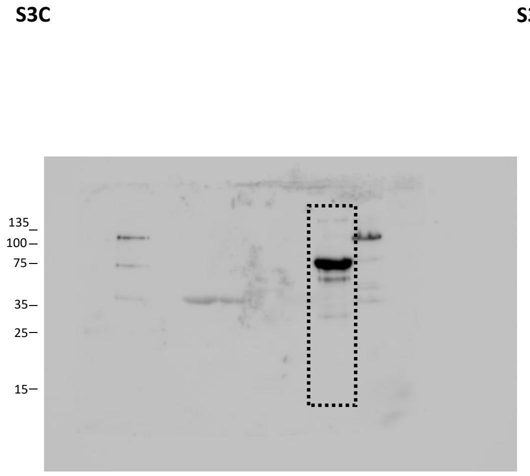

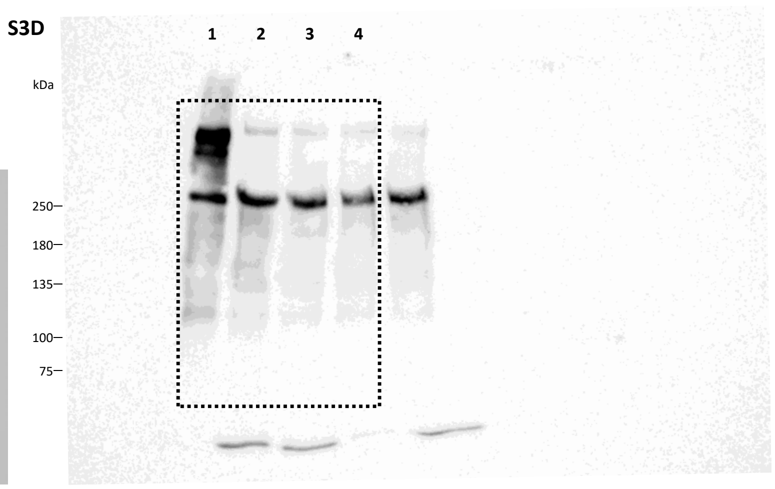

1: vWF
2: SH1000 WT + vWF
3: SH1000 *spA* + vWF
4: SH1000 *isdB* + vWF

Fig S3 – Original full-length western blots corresponding to the main figures shown in the manuscript. The cropped area used in the manuscript is shown by a dotted line frame in the original full western blots. *A* (Fig. 1A in the manuscript). *B* (Fig. 1B in the manuscript). *C* (inset of Fig. 1C in the manuscript). *D* (Fig. 2A in the manuscript).
